# CpG methylation as a tool to characterize cell-free Epstein-Barr virus DNA

**DOI:** 10.1186/1750-9378-7-S1-P29

**Published:** 2012-04-19

**Authors:** Meir Shamay, Nicholas Hand, Richard F Ambinder

**Affiliations:** 1 Department of Oncology, Johns Hopkins School of Medicine, Baltimore, MD, USA

## Background

In order to differentiate Epstein-Barr virus (EBV) virion DNA versus viral DNA released from tumor cells, we have taken advantage of the observation that viral episomal genomes of herpesviruses are methylated in latently infected cells whereas un-methylated genomes are synthesized and packaged into virions during the lytic phase. We used paramagnetic beads linked to methylCpG binding protein to separate virion and cell-derived viral DNA.

**Figure 1:**
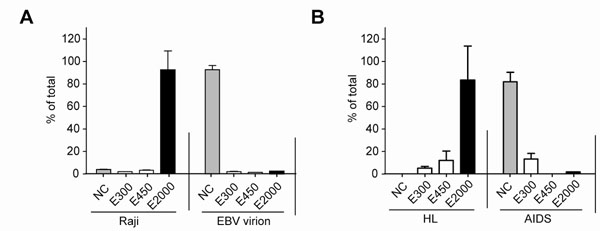
(A) DNA isolated from purified EBV virions or from latently infected Raji cells were subjected to binding to paramagnetic beads linked to methylCpG binding protein. DNA isolated from the non-captured fraction (NC), washes (300mM and 400mM) and the elution (2000mM) was subjected to real-time PCR with primers that amplify a region in EBV BamW. (B) DNA isolated from the plasma of an AIDS patient and the plasma of a patient with EBV(+) Hodgkin lymphoma was subjected to binding to paramagnetic beads linked to methylCpG binding protein. DNA isolated from the different fractions was amplified as in B.

DNA isolated from EBV (Figure 1A) virions failed to bind to the methylCpG binding protein and were detected only in the non-captured (NC) fractions, while DNA isolated from latently infected cell lines were detected predominantly in the bound fractions ( E2000, high salt elute). Unmethylated EBV DNA, presumably virion DNA, was detected in the plasma of 3 AIDS patients without lymphoma, while methylated DNA was detected in the blood of 3 patients with EBV-associated Hodgkin lymphoma (HL) (without HIV infection) (Figure 1B).

## Conclusions

Tumor derived viral DNA can be distinguished from virion associated viral DNA based on preferential binding to methylCpG binding protein. Tumor derived viral DNA was predominantly present in the blood from patients with Hodgkin-Lymphoma, but not in patients without EBV associated malignancy. This technique may be applied to detect tumor derived viral DNA in the blood of patients with EBV associated malignancies.

